# Prevalence of abnormal spirometry and correlates of lung function decline among a population in rural Uganda

**DOI:** 10.7189/jogh.16.04267

**Published:** 2026-10-02

**Authors:** Marvah Hill Pierre-Louis, Yiqi Qian, Emily N Satinsky, Edwin Nuwagira, Charles Baguma, Patience Ayebare, Patrick Gumisiriza, Megan Lam, Mi-Sun Lee, David C Christiani, Stephen Asiimwe, Bernard Kakuhikire, Alexander C Tsai, Mark J Siedner, Crystal M North

**Affiliations:** 1 Pulmonary and Critical Care Medicine, Massachusetts General Hospital, Boston, Massachusetts, USA; 2 Medical Practice Evaluation Center, Massachusetts General Hospital, Boston, Massachusetts, USA; 3 Center for Global Health, Massachusetts General Hospital, Boston, Massachusetts, USA; 4 Mbarara University of Science and Technology, Mbarara, Uganda; 5 Harvard T.H. Chan School of Public Health, Boston, Massachusetts, USA; 6 Harvard Medical School, Boston, Massachusetts, USA; 7 Division of Infectious Diseases, Massachusetts General Hospital, Boston, Massachusetts, USA; 8 Africa Health Research Institute, KwaZulu-Natal, South Africa

**Keywords:** chronic lung disease, COPD, asthma, pulmonary function testing, resource-limited setting(s)

## Abstract

**Background:**

Chronic lung disease, which is the third leading cause of death globally, places a disproportionate burden of morbidity and mortality on sub-Saharan Africa, where limited infrastructure results in a lack of understanding of how lung function changes over time, whether it varies among susceptible populations, or whether at-risk individuals could be identified before abnormal lung function develops. To address this gap, we aimed to characterise lung function patterns and identify correlates of lung function decline in rural Uganda.

**Methods:**

Adults completed health questionnaires and American Thoracic Society/European Respiratory Society-acceptable spirometry up to three times over four years (2015–2019). Our primary outcome was lung function (forced expiratory volume in 1 second (EV_1_) and FEV_1_/forced vital capacity (FVC)); our secondary outcome was spirometry patterns. We characterised baseline patterns using Global Lung Initiative (GLI)-Global prediction equations and identified correlates of decline using generalised mixed effects linear regression. We then explored effect modification by sex, smoking, and socioeconomic status using interaction terms, with inverse probability of treatment weights balancing baseline characteristics.

**Results:**

A total of 902 participants completed 1,471 ATS/ERS-acceptable spirometry tests. Their median age was 35 years, 56% were women, 75% were never-smokers, and 31% experienced chronic respiratory symptoms. Abnormal lung function affected 14% of the cohort, more commonly women and those with respiratory symptoms. FEV_1_ declined by 10mL/year (95% CI = −17, −2), and FEV_1_/FVC declined by 0.006/year (95% CI = −0.008, −0.005) or 0.53% predicted/year (95% CI = −0.68, −0.37). Among those with normal baseline lung function, FEV_1_ and FEV_1_/FVC declined more rapidly (FEV_1_ −18mL/year; FEV_1_/FVC −0.66% predicted). There was no effect modification by sex, smoking, or SES.

**Conclusions:**

Lung function decline may be more rapid in this young, mostly non-smoking population. Our findings extend the understanding of non-obstructive lung disease in sub-Saharan Africa and emphasise that risk persists despite normal spirometry. These data can inform regional public health strategies for risk mitigation, early disease detection, and building healthcare infrastructure for the growing population at risk for or living with chronic lung disease.

Chronic lung disease is the third leading cause of death globally, responsible for 3.9 million deaths and 112 million disability-adjusted life years annually [1]. Chronic obstructive pulmonary disease (COPD) and asthma are the most prevalent chronic lung diseases, affecting 480 million and 262 million people globally, respectively [2,3]. More recently, additional patterns of abnormal spirometry, referred to as preserved ratio impaired spirometry (PRISm) and restrictive spirometry pattern have been the subject of increased investigations, as these patterns of abnormal spirometry are associated with incident respiratory and cardiovascular diseases, and increased morbidity and mortality [4,5].

The prevalence, risk factors, and morbidity and mortality of chronic lung disease are known to vary by region and gender. For example, the BOLD study, which is among the first global studies of lung disease burden, estimated an obstructive lung disease prevalence among men ranging from 6% in India to 23% in South Africa and among women from 4% in the Philippines to 21% in Sweden [6]. A recent systematic review and meta-analysis estimated that the prevalence of COPD in sub-Saharan Africa is 8% [7], which is higher than estimates for the USA [8] and Europe [9]. Indeed, by 2050, sub-Saharan Africa is projected to have the highest COPD prevalence in the world [2]. Similarly, there are 120 million people living with asthma in sub-Saharan Africa [10], nearly a third of whom may meet the criteria for severe asthma [11]. There is also regional variation in the influence of socioeconomic status (SES) on the burden of lung disease [6]. In high income countries, lower SES was found to be associated with poorer lung health [12]. Comparatively, data on the relationship between SES and lung health in low-and-middle-income countries (LMICs) are quite limited [13], with studies showing both higher [14] and lower [15] respiratory morbidity among those of lower SES.

Smoking is the leading risk factor for chronic lung disease among men and in high income countries, while air pollution is the leading risk factor for lung disease among women and in LMICs [16]. Tuberculosis and chronic HIV infection, common conditions in many LMICs, were also found to be associated with higher risk of chronic lung diseases and are important drivers of non-smoking chronic lung disease risk [17,18]. The prevalence, costs, and morbidity and mortality of chronic lung disease have rapidly increased over the last 30 years – disproportionately so in LMICs and particularly among women [1,16,19].

However, diagnosing lung disease requires equipment that is not widely available in many LMICs. Given the limited infrastructure available for tracking population lung health over time in these regions, few cohorts have attempted to characterise longitudinal lung health, leaving us with limited knowledge regarding how lung function changes over time, whether it varies among particularly susceptible populations, or whether at-risk populations could be identified prior to the development of abnormal lung function. To address these knowledge gaps, we measured serial lung function over four years within a well-characterised cohort of 902 adults living in rural Uganda to characterise lung function patterns and identify correlates of lung function decline.

## METHODS

Adults who reported stable primary residence in Nyakabare Parish in southwestern Uganda were enumerated in a population census from 2014 to 2015 [20], with parish residents who were at least 18 years of age invited to attend free health screening events that occurred in 2015, 2017, and 2019. Each health screening event lasted five days and was geographically distributed across three locations in the parish to ensure easy accessibility for all residents (Figure S1 in the **Online Supplementary Document**) [21]. At each health screening event, parish residents could consent to have their health information collected for research purposes. We included those who were at least 18 years old, attended at least one health screening event, and consented to have their health information collected for research purposes in this analytical cohort. We otherwise set no other exclusion criteria.

At each health screening event, participants completed health history questionnaires that included demographics, SES, medical history, smoking history, and respiratory symptoms. Study staff measured participants’ height using a stadiometer and weight using electronic scales, and body mass index (BMI) was calculated as weight in kilograms divided by the square of height in meters. We measured SES through a principal components analysis based upon a household asset index, in which ownership of more household assets defines greater wealth and thus higher SES [22]. Then, we created quintiles of asset ownership (and thus SES) from the asset ownership index, in which quintile 1 represented those with the most assets (*i.e.* the ‘richest’ quintile) and quintile 5 represented those with the least assets (*i.e.* the ‘poorest’ quintile). Chronic cough was defined as the presence of a cough on most days; for those diagnosed with chronic cough, we ascertained the frequency of sputum production. Chronic dyspnoea was defined as having difficulty breathing on most days.

Participants completed spirometry with the guidance of study staff using EasyOne® Plus handheld spirometers (nDD Medical Technologies, Andover, Massachusetts, USA) in accordance with American Thoracic Society/European Respiratory Society (ATS/ERS) standards. Spirometry was considered ATS/ERS acceptable if the forced expiratory volume in 1 second (FEV_1_) and forced vital capacity (FVC) from the two best manoeuvres were within 200 mL of one another. Per automated spirometer algorithms, participants with FEV_1_/FVC<0.7 completed repeat spirometry 10 minutes after receiving four puffs of albuterol (Ventolin, GlaxoSmithKline, Philadelphia, Pennsylvania, USA), the results of which we interpreted using Global Lung Initiative (GLI)-Global prediction equations and ATS/ERS interpretation guidelines [23,24] and only ATS/ERS-acceptable tests were included in the analytic cohort. For those with ATS/ERS acceptable post-bronchodilator spirometry, COPD was defined as a post-bronchodilator FEV_1_/FVC < lower limit of normal (LLN) [23,24].

### Statistical analysis

The primary outcome of interest was change in lung function (pre-bronchodilator FEV_1_ and FEV_1_/FVC), while the secondary outcome of interest was prevalence of abnormal spirometry, which included obstruction, spirometric restriction, and PRISm. We chose not to focus on change in FVC over time because FVC is more effort- and time-dependent than other spirometry metrics in field studies and therefore less reproducible over time [25,26].

To evaluate for selection bias, we compared baseline demographics between participants who had completed ATS/ERS-acceptable *vs*. unacceptable spirometry, and between participants in our final analytic cohort *vs*. participants excluded from our final analytic cohort. We tested all subgroup differences using Wilcoxon rank sum, chi-squared, or Fisher’s exact tests, as appropriate.

Similar to our prior work [21], we used inverse probability of treatment (IPT) weights to estimate population-representative estimates by reducing selection bias. We first estimated the propensity for parish adults to attend at least one health screening event using a logistic regression model consisting of census variables anticipated to correlate with event attendance. Variables included age, sex, education, village, self-reported overall health, and SES. Using this model, we calculated stabilised IPT weights [27] and incorporated these weights in our regression models.

We then summarised baseline demographics for the whole cohort and by sex, and the proportion of study participants with chronic respiratory symptoms and abnormal lung function, both with and without the weights. We considered each spirometry metric as an absolute value and as a percent predicted value based on GLI-Global prediction equations [24]. We categorised lung function as obstruction (FEV_1_/FVC<LLN) [23,28], spirometric restriction (FEV_1_/FVC ≥ LLN with FEV_1_ and FVC both < LLN), and preserved ratio impaired spirometry (PRISm; FEV_1_/FVC ≥ LLN with FEV_1_ < 80% predicted) [23]. We chose our covariates of interest based on established associations with lung function, which included age, sex, height, smoking, occupation, education, and SES. We dichotomised occupation based upon the most prevalent self-reported occupations (subsistence farmers *vs*. all other employment types).

We then modelled lung function trajectories using IPT-weighted generalised mixed effects linear regression models adjusted for age at baseline, sex, height, smoking, occupation, education, and SES. Models accounted for intra-cluster correlations within participants and allowed for subject-specific baseline lung function using random intercepts. We evaluated for evidence of effect modification on the change in lung function over time by sex, smoking, and SES (dichotomised at median asset wealth) by including *effect modifier* × *time* product terms. Given that lung function increases until about 25 to 30 years of age [29], we evaluated the robustness of our results in sensitivity analyses restricted to participants at least 30 years of age and 40 years of age. To further test the robustness of our results, we performed additional sensitivity analyses excluding those with abnormal lung function at cohort entry. We applied a Bonferroni correction to provide strong control of familywise error (two lung function outcomes: *P* = 0.05/2 = 0.025 for). We conducted statistical analyses using SAS, version 9.4 (SAS Institute, Carey, North Carolina, USA), *R*, version 4.4.1 (R Core Team, Vienna, Austria) and Stata, version 17 (StataCorp LLC, College Station, Texas, USA).

## RESULTS

Of the 2,245 adults living in Nyakabare Parish enumerated in the 2015, 2017, or 2019 population census, 1,246 (56%) attended at least one health screening event. Of the 1219 (98%) participants who had completed spirometry at least once during the four-year study period, 902 (74%) completed ATS/ERS-acceptable spirometry, had complete information on regression model covariates, and were thus included in the analytic cohort (Figure S2 in the **Online Supplementary Document**). Just over half of participants with ATS/ERS-acceptable spirometry (n = 479, 53%) attended only one health screening event, 277 (31%) attended two health screening events, and 146 (16%) attended all three health screening events. Participants who had completed ATS/ERS-acceptable spirometry were more educated, had higher BMI, and more commonly worked as subsistence farmers than participants who did not complete ATS/ERS-acceptable spirometry (Table S1 in the **Online Supplementary Document**). Participants in the final analytic cohort were slightly older, more educated, more commonly worked as subsistence farmers, and had higher BMI than participants excluded from the final analytic cohort (Table S2 in the **Online Supplementary Document**).

Using IPT weights to estimate population-level data (Figure S3, Panels A and B in the **Online Supplementary Document**), the baseline median age was 35 years old (interquartile range (IQR) = 26, 49), 56% were women, most of the population (75%) had never smoked, and 60% completed at least a primary school education (**Table 1**). Most participants had either a normal BMI (59%) or were overweight (25%), and subsistence farming was the most common occupation (65%). Compared to men, women smoked less (*P* < 0.001), were less educated (*P* < 0.001), were more commonly subsistence farmers (*P* < 0.001), and had higher BMI (*P* < 0.001). Among those who had completed health history questionnaires, few had a known diagnosis of asthma (2%) or COPD (1%).

**Table 1:** Baseline cohort characteristics and population characteristics*

	Total cohort (n = 902)	Weighted estimates	Women (n = 557)	Weighted estimates	Men (n = 345)	Weighted estimates	*P-*value†
**Age in years, Mdn (IQR)**	40 (29, 53)	35 (26, 49)	38 (29, 51)	35 (27, 49)	42 (31, 54)	36 (26, 49)	0.553
**Women**	557 (62)	56	557 (100)	100	0 (0)	0	<0.0001
**Smoking history**							<0.0001
Never	640 (71)	75	449 (81)	84	191 (56)	63	
Former	175 (20)	16	89 (16)	13	86 (25)	20	
Current	82 (9)	9	16 (3)	3	66 (19)	17	
**Years smoked, Mdn (IQR)‡**	21 (10, 32)	21 (9, 32)	7 (2, 10)	7 (2, 10)	25 (11, 33)	23 (10, 32)	0.0002
**Education**							<0.0001
None	124 (14)	11	99 (18)	16	25 (7)	6	
Some primary school	299 (33)	28	196 (35)	30	103 (30)	26	
Completed primary school	236 (26)	25	131 (24)	22	105 (30)	29	
At least some secondary school	243 (27)	35	131 (24)	32	112 (32)	39	
**Subsistence farming**	628 (71)	65	445 (81)	78	183 (53)	48	<0.0001
**Socioeconomic status**							0.094
Poorest	186 (21)	20	124 (22)	22	62 (18)	18	
Poorer	183 (20)	19	114 (21)	19	69 (20)	19	
Average	182 (20)	20	118 (21)	22	64 (19)	19	
Richer	192 (21)	20	116 (21)	20	76 (22)	21	
Richest	157 (17)	20	83 (15)	17	74 (21)	24	
**BMI in kg/m^2^**							<0.0001
Underweight (<18.5)	53 (6)	5	20 (4)	2	33 (10)	8	
Normal (18.5–25)	498 (55)	59	241 (43)	24	257 (74)	77	
Overweight (25.1–30)	235 (26)	25	192 (34)	34	43 (12)	12	
Obese (≥30)	116 (13)	12	104 (19)	18	12 (3)	3	
**Medical comorbidities**							
COPD	4 (1)	1	1 (0)	0	3 (2)	1	0.247
Asthma	9 (2)	2	5 (1)	1	4 (2)	2	0.314
HIV	25 (5)	4	17 (6)	5	8 (4)	3	0.453
Diabetes	14 (3)	2	8 (2)	2	6 (3)	2	0.943

BMI – body mass index, COPD – chronic obstructive pulmonary disease, IQR – interquartile range, Mdn – median

*Presented as n (%) or % (for weighted estimates) unless specified otherwise. Data were missing for ≤5% of participants for all covariates other than the following: COPD: 371 participants (41%); asthma: 364 participants (40%); HIV: 423 participants (47%); diabetes: 364 participants (40%). For these covariates, the above percentage distributions are restricted to participants without missing data.

†Continuous variables were compared using Wilcoxon rank-sum tests. Categorical variables were compared using chi-squared tests. All comparisons were weighted.

**‡**Among current or former smokers only.

Respiratory symptoms were common (**Table 2**). Nearly a third (31%) of study participants reported chronic cough or chronic dyspnoea, both of which were more common among women than men (*P* = 0.002 and *P* = 0.021, respectively). Nearly two thirds (63%) of those with chronic cough reported sputum production on most days. In terms of baseline lung function, median FEV_1_ was 2607 mL (interquartile range (IQR) = 2150, 3152), median FVC was 3212 mL (IQR = 2,712, 3,817), and median FEV_1_/FVC was 0.82 (IQR = 0.78, 0.86). About 86% of the study cohort had normal spirometry throughout the study period, which was less common among women than men (83% *vs*. 91%; *P* = 0.002) and among those with *vs*. without respiratory symptoms (78% *vs*. 91%; *P* < 0.0001). The proportion of the study population with obstruction (7%), restriction (1%), and PRISm (1%) did not differ between women and men, but the proportion of the population with combined restriction and PRISm was significantly higher among women than men (6% *vs*. 1%; *P* = 0.0002). Among those with pre-bronchodilator obstruction, 84% completed ATS/ERS-acceptable post-bronchodilator spirometry, of whom 44% had post-bronchodilator obstruction, with no difference between men and women (50% *vs*. 39%; *P* = 0.432).

**Table 2:** Baseline unadjusted and population-weighted respiratory health*

	Total cohort (n = 902)	Weighted estimates	Women (n = 557)	Weighted estimates	Men (n = 345)	Weighted estimates	*P*-value†
**Respiratory symptoms**							
Any symptoms‡	290 (32)	31	206 (37)	37	84 (25)	23	<0.0001
Chronic dyspnea	157 (18)	17	109 (20)	20	48 (14)	13	0.021
Chronic cough	208 (23)	22	144 (26)	26	64 (19)	17	0.002
Sputum production§	123 (61)	63	78 (56)	58	45 (71)	73	0.052
**Lung function**							
FEV_1_, mL	2,469 (2,048, 2,988)	2,607(2,150, 3152)	2,256 (1,900, 2,553)	2,309 (1,942, 2,607)	3,040 (2,612, 3501)	3,195 (2,712, 3,587)	<0.0001
FVC, mL	3,082 (2,584, 3,648)	3,212 (2,712, 3,817)	2,766 (2,374, 3,131)	2,810 (2,430, 3,169)	3,800 (3,346, 4,200)	3,849 (3,441, 4,244)	<0.0001
FEV_1_/FVC	0.81 (0.77, 0.85)	0.82 (0.78, 0.86)	0.81 (0.77, 0.85)	0.82 (0.78, 0.85)	0.81 (0.76, 0.85)	0.83 (0.78, 0.86)	0.164
Normal spirometry	772 (86)	86	461 (83)	83	311 (90)	91	0.002
Obstruction only¶	66 (7)	7	44 (8)	8	22 (6)	6	0.393
Restriction only	7 (1)	1	2 (0)	0	5 (1)	1	0.113
PRISm only¶	14 (2)	1	12 (2)	2	2 (1)	0	0.063
PRISm + restriction¶	43 (5)	4	38 (7)	6	5 (1)	1	0.0002

ATS – American Thoracic Society, COPD – chronic obstructive pulmonary disease, FEV_1_ – forced expiratory volume in one second, FVC – forced vital capacity, IQR – interquartile range, LLN – lower limit of normal, Mdn – median, PRISm – preserved ratio impaired spirometry

*Presented as n (%) or % (for weighted estimates) unless specified otherwise. Data were missing for ≤5% of participants for all covariates. The above percentage distributions are restricted to participants without missing data. Unweighted percentages may not sum to 100% due to rounding. Weighted percentages reflect the individual-level sampling weights and may not sum exactly to 100%.

†Continuous variables were compared using Wilcoxon rank-sum tests. Categorical variables were compared using chi-squared tests. *P*-values compare weighed estimates among women *vs*. men.

‡Defined as reporting chronic dyspnea or chronic cough.

§Ascertained only among those with chronic cough, and the proportion represents the proportion of participants with chronic cough who reported sputum production on most days.

¶Obstruction and PRISm were defined using FEV_1_/FVC<LLN criteria

In adjusted models, FEV_1_ declined by −10mL/year (95% confidence interval, CI = −17, −2, *P* = 0.009) and FEV_1_/FVC by −0.006/year (95% CI = −0.008, −0.005, *P* < 0.0001). In models using percent predicted values, FEV_1_/FVC declined by −0.53% predicted/year (95% CI = −0.68, −0.37, *P* < 0.0001), while the yearly decline in FEV_1%_ predicted was not statistically significant (**Table 3**; Figure S3 and Table S3 in the **Online Supplementary Document**). Interestingly, when restricting the analytic cohort to the 772 individuals with normal lung function at cohort entry, FEV_1_ and FEV_1_/FVC declines were even more rapid than the those observed in the whole cohort (FEV_1_ = −18mL/year; FEV_1_/FVC = −0.66% predicted (Table S4 in the **Online Supplementary Document**). As expected, in the sensitivity analyses limited to older individuals, the annual FEV_1_ decline was the more rapid (*e.g.* annual FEV_1_ decline = −20mL/year among those at least 40 years old), although the FEV_1_/FVC decline was unchanged (Table S5 in the **Online Supplementary Document**). We found no evidence for effect modification on the change in lung function over time by sex, smoking, or SES (**Figure 1**, **Table 3**).

**Table 3:** Population-weighted annual lung function decline among adults living in rural Uganda (n = 902)*

	ΔFEV_1_ (mL)		ΔFEV_1_ (% pred)		ΔFEV_1_/FVC		ΔFEV_1_/FVC (% pred)	
	***β* (95% CI)**	***P*-value**	***β* (95% CI)**	***P*-value**	***β* (95% CI)**	***P*-value**	***β* (95% CI)**	***P*-value**
**Change per year**	−10 (−17, −2)	0.01	0.28 (−0.02, 0.55)	0.05	(−0.008, −0.005)	<0.001	−0.53 (0.68, −0.37)	<0.001
**Lung function trajectory (per study visit) in stratified models**								
	***β* (95% CI)**		***β* (95% CI)**		***β* (95% CI)**		***β* (95% CI)**	
**Sex**								
Women	−12 (−21, −3)	0.01	0.17 (−0.19, 0.52)	0.36	−0.007 (−0.008, −0.005)	<0.001	−0.56 (−0.76, −0.37)	<0.001
Men	−6 (−18, 6)	0.36	0.48 (0.003, 0.95)	0.05	−0.006 (−0.008, −0.004)	<0.001	−0.46 (−0.72, −0.20)	0.001
*P*-value for interaction	0.42		0.31		0.36		0.55	
**Smoking**								
Ever smokers	−9 (−24, 6)	0.26	0.45 (−0.13, 1.03)	0.13	−0.005 (−0.008, −0.003)	<0.001	−0.50 (−0.81, −0.18)	0.002
Never smokers	−10 (−19, −2)	0.02	0.22 (−0.11, 0.55)	0.19	−0.007 (−0.008, −0.005)	<0.001	−0.54 (−0.72, −0.36)	<0.001
*P*-value for interaction	0.85		0.51		0.38		0.82	
**SES**								
Lower SES	−1 (−13, 10)	0.82	0.58 (0.15, 1.02)	0.01	−0.005 (−0.007, −0.004)	<0.001	−0.40 (−0.64, −0.16)	0.001
Higher SES	−17 (−27, −7)	0.001	0.04 (−0.35, 0.43)	0.86	−0.007 (−0.009, −0.005)	<0.001	−0.63 (−0.85, −0.42)	<0.001
*P*-value for interaction	0.05		0.07		0.20		0.15	

CI – confidence interval, FEV_1_ – forced expiratory volume in one second, in millilitres, FVC – forced vital capacity, in millilitres, SES – socioeconomic status, *β* – beta coefficient

*All models adjusted for age at baseline, sex, height, smoking, occupation, education, and SES (dichotomised at median wealth based on asset ownership index).

**Figure 1:**
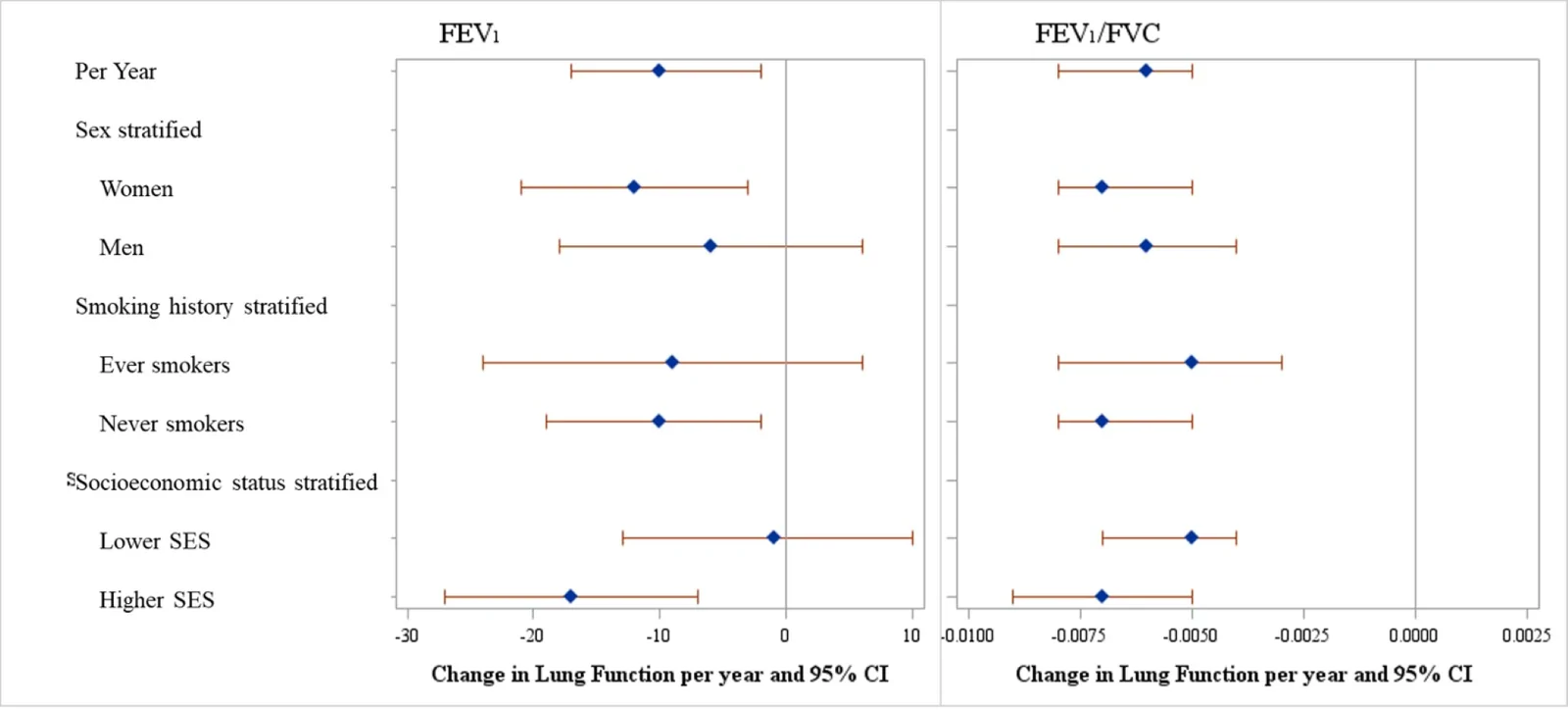
Forest plot of change in lung function per visit and 95% CI among adults living in rural Uganda (n = 902). CI – confidence interval, FEV_1_ – forced expiratory volume in one second, in milliliters, FVC – forced vital capacity, in milliliters, SES – socioeconomic status.

FEV_1_ and FEV_1_/FVC were lower among older adults (per additional year in age: FEV_1_ = −20mL; 95% CI = −22, −18; FEV_1_/FVC = −0.002; 95% CI = −0.002, −0.002), among women *vs*. men (FEV_1_ = −559 mL; 95% CI = −629, −488; FEV_1%_ predicted = −7.45; 95% CI = −10.23, −4.67; FEV_1_/FVC % predicted = −1.48; 95% CI = −2.79, −0.16), and among ever smokers *vs*. never smokers (FEV_1_/FVC = −0.010; 95% CI = −0.018, −0.002; FEV_1_/FVC % predicted = −1.28; 95% CI = −2.26, −0.30). Relationships between SES and lung function metrics were not statistically significant (Table S3 in the **Online Supplementary Document**).

## DISCUSSION

In this population-based cohort of 902 mostly non-smoking young adults in rural Uganda who had completed 1,471 ATS/ERS-acceptable spirometry tests across four years, we found that nearly a third of the population reported chronic respiratory symptoms and 14% had abnormal spirometry, both of which were more common among women. We also found that the annual FEV_1_ decline was similar to published estimates [30], while the FEV_1_/FVC declined more rapidly than published estimates, with no evidence of effect modification by sex, smoking, or SES. Importantly, participants with normal lung function at cohort entry had an even more rapid FEV_1_ and FEV_1_/FVC decline compared to the whole cohort. The coexistence of prevalent respiratory symptoms and a more rapid FEV_1_ and FEV_1_/FVC decline in this relatively young rural population, particularly among those with normal lung function on initial testing, highlights a vulnerable population at increased future risk of lung disease. Our results add to the literature in several important ways.

There are only a few other population-based longitudinal studies of lung function in sub-Saharan Africa to which we can compare our results. Among 675 adults in Malawi who had completed spirometry twice over five years, FEV_1_ decline was 46.1mL/year, while the FEV_1_/FVC decline was not reported in the article [31]. The participants in the Malawian cohort were generally older than those in our study, and since lung function decline accelerates with age [32], this may explain the faster FEV_1_ decline in the Malawi study. However, FEV_1_ decline among mostly non-smoking older adults in the USA, Europe, and Australia ranged from 9.9 to 56 mL/year [32], suggesting that the lower FEV_1_ decline in our cohort may represent regional variation across Africa. To date, the Framingham Offspring Cohort is the only other study to have reported on the population-level longitudinal FEV_1_/FVC decline, placing it at 0.0029/year over a median of 17 years among 1,332 participants [33], which half that of our cohort estimate. This is an important finding because rapid FEV_1_/FVC decline was found to be associated with incident COPD and higher respiratory and all-cause mortality [34]. Furthermore, nearly one third of our participants reported chronic respiratory symptoms, most of whom had normal lung function, which is consistent with our prior work [21]. While it is possible that those with respiratory symptoms have undiagnosed parenchymal lung disease, respiratory symptoms with normal spirometry could be associated with incident COPD [35] and may represent an increasingly recognised risk group identified as ‘pre-COPD’ [36], meaning they are recognised to be at a crucial time point at which interventions to prevent disease could be the most influential. Indeed, the lung function decline in our cohort was the fastest among those with normal lung function. Together, these results emphasise that this population of Ugandan adults with mostly normal lung function carries significant risk of future respiratory morbidity and mortality.

Our lower-than-anticipated estimates of the prevalence of non-obstructive abnormal spirometry and the higher prevalence among women than men add to a small but growing body of literature characterising non-obstructive spirometry patterns in sub-Saharan Africa, though direct comparisons to our results are challenged by variations in spirometry prediction equations. In a cross-sectional analysis of 3,580 Ugandan adults, the prevalence of PRISm (defined using a fixed FEV_1_/FVC ratio) was 16%, with higher odds of the pattern occurring among women as compared to men [37]. Among 749 Malawian adults, restrictive spirometry pattern (defined using locally derived prediction equations) was present among 9% of the sample, yet no data were provided on differences by sex [38]. In Cameroon, 18.8% of 1,003 adults were found to have restrictive spirometry pattern, also defined using locally derived prediction equations [39]. In the BOLD study, in which the authors used NHANES III Caucasian prediction equations to define spirometry abnormalities, restrictive spirometry prevalence was 46.5% in South Africa, 47.6% in Malawi, and 70.7% in Nigeria [40]. Lastly, among 339 Ugandan adults who had recovered from pneumonia, 31% had PRISm, with the odds of the pattern occurring being higher among women [41]. The higher prevalence of PRISm in this Ugandan cohort compared to ours likely stems from the entirety of the cohort having had pneumonia. In our study, abnormal spirometry was more common among women than men – a difference driven largely by more prevalent combined PRISm and restriction. Whether this observation relates to differences in exposures (*e.g.* more air pollution), susceptibility to lung disease, or a combination therein is an area of active investigation by our group and others. Furthermore, it may be the case that the lower-than-anticipated PRISm and restrictive spirometry prevalence estimates in the current study are due to the use of different lung function prediction questions, as was demonstrated in a Nigerian population [42], or the use of LLN thresholds to define normal lung function. In the future, standardised recommendations for the use of LLN thresholds to define normal lung function among younger populations could facilitate comparisons across study cohorts.

Several of our findings are consistent with the published literature. For example, our estimates for the prevalence of obstruction are consistent with COPD estimates in Uganda, which range from 1.7% to 16% [21,43–45]. Our estimates, however, are higher than those put forward by prior cross-sectional work by our group among a subset of the current study population, in which obstruction prevalence was 2.3% [21]. In this prior work, we defined COPD using a fixed FEV_1_/FVC ratio, which underestimates obstruction in younger populations. Although annual lung function decline did not differ among sub-populations, we did find cross-sectional FEV_1_ and FEV_1_/FVC to be lower among older individuals and among women, and FEV_1_/FVC to be lower among current/former smokers, all of which is consistent with published literature [32,46,47].

Lastly, our results emphasise the importance of non-smoking exposures and lung disease in East Africa. Abnormal lung function was prevalent despite three quarters of the population having never smoked. Furthermore, those who had never smoked had similar lung function decline as current/former smokers, which may reflect the low pack-years among current/former smokers in the region [48]. These results are consistent with recent global estimates suggesting that smoking is not the main driver of lung disease in LMICs and highlight the importance of focusing on regional drivers of lung function decline, such as air pollution and post-tuberculosis lung disease [49,50]. Work to reduce lung disease risk among at-risk populations must focus on non-smoking risk factors to appropriately target the highest risk exposures in the region.

The strength of this study lies in its prospective design, the population-based sample in which IPT weighting was used to reduce selection bias, and the use of spirometry to objectively measure lung function. Our study also has some limitations. Participants chose to participate in the study, even though this meant they had to organise their own transportation to the free health screening event. This may have resulted in a healthier cohort, though this risk was offset with IPT weights to reduce bias. Not all participants completed ATS/ERS-acceptable post-bronchodilator spirometry, so we were unable to differentiate between COPD and asthma among those with pre-bronchodilator obstruction. In the absence of body plethysmography, we were unable to measure total lung capacity and thus could not confirm that the restrictive spirometry pattern represented restrictive lung disease. HIV serostatus and prior tuberculosis are both important drivers of chronic lung disease in sub-Saharan Africa [51,52], but were missing among nearly half of the study population. Furthermore, among those with non-missing values, these data points were collected by voluntary self-report, as neither confirmatory testing (for HIV) nor national treatment cards (for tuberculosis) were available. As such, we were unable to evaluate the extent to which chronic HIV infection or post-tuberculosis lung disease contribute to abnormal lung function patterns or lung function decline in rural Uganda. There were also no data available on environmental risks such as air pollution exposure, which limits our ability to explore the extent to which air pollution exposure influences lung function decline. With that said, our prior work confirms that prior TB is associated with subsequent lung dysfunction [50,53], that people with HIV have accelerated loss of lung function [54], that most people in the study region use biomass fuels for cooking and that regional air pollution is higher than international standards [49,55]. Lastly, as a single-region study, our results may not be generalisable beyond the East African setting.

## CONCLUSIONS

Respiratory symptoms are common and lung function decline may be more rapid in this population of young, mostly non-smoking adults in rural Uganda. This work extends our understanding of non-obstructive lung disease burden in sub-Saharan Africa and highlights that lung disease risk persists despite normal spirometry. These data can inform public health strategies focused on risk mitigation, early disease detection, and developing the requisite healthcare infrastructure for the growing population of people with chronic lung disease in sub-Saharan Africa.

## Additional material


Online Supplementary Document

## Data Availability

**Data availability**: De-identified data can be made available upon reasonable request to the corresponding author.
