# Supplementary material for: Prevalence of abnormal spirometry and correlates of lung function decline among a population in rural Uganda
Source: J Glob Health. 2026 Oct 2;16:04267. doi: 10.7189/jogh.16.04267 (PMC13631771; doi:10.7189/jogh.16.04267)
Supplement: Supplementary file 1 [file jogh-16-04267-s001.pdf]

Supplement to: Pierre-Louis MH, Qian Y, Satinsky EN, Nuwagira E, Baguma C, Ayebare P, Gumisiriza P, Lam M, Lee MS, Christiani DC, Asiimwe S, Kakuhikire B, Tsai AC, Siedner MJ, North CM. Prevalence of abnormal spirometry and correlates of lung function decline among a population in rural Uganda. J Glob Health. 2026;16:04267.

Table S1. Baseline cohort characteristics among participants who completed ATS acceptable spirometry at least once compared to participants without any ATS acceptable spirometry

	Total Cohort (n = 1219)	ATS acceptable (n = 946)	Not ATS acceptable (n = 273)	P value[#]
**Age**, years	38 [28, 53]	39 [29, 52]	36 [25, 53]	0.083
**Women**	733 (60)	570 (60)	163 (60)	0.871
**Smoking history**				0.402
Never	850 (70)	668 (71)	182 (67)	
Former	242 (20)	183 (20)	59 (22)	
Current	116 (10)	86 (9)	30 (11)	
Years smoked	18 [8, 32]	21 [10, 32]	7 [3, 12]	0.034
**Education**				0.0007
None	185 (15)	127 (14)	58 (22)	
Some primary school	390 (33)	306 (33)	84 (32)	
Completed primary school	296 (25)	250 (27)	46 (17)	
At least some secondary school	327 (27)	251 (27)	76 (29)	
**Subsistence farming**	794 (69)	636 (71)	158 (62)	0.017
**Socioeconomic status**				0.166
Poorest	261 (22)	195 (21)	66 (26)	
Poorer	244 (21)	186 (20)	58 (22)	
Middle	233 (20)	189 (20)	44 (17)	
Richer	236 (20)	195 (21)	41 (16)	
Richest	213 (18)	164 (18)	49 (19)	
**Body Mass Index**, kg/m^2				0.001
Underweight (<18.5)	80 (7)	56 (6)	24 (9)	
Normal (18.5–25)	701 (58)	528 (56)	173 (63)	
Overweight (25.1–30)	305 (25)	243 (26)	62 (23)	
Obese (≥30)	133 (11)	119 (13)	14 (5)	
**Medical Comorbidities**				
COPD	7 (1)	4 (0)	3 (1)	0.355
Asthma	9 (1)	9 (1)	0 (0)	0.227
HIV	34 (3)	25 (3)	9 (3)	0.724
Diabetes	23 (2)	14 (1)	9 (3)	0.141

n (%) or median [interquartile range] unless otherwise specified.

[#] Continuous variables: Wilcoxon rank sum tests; Categorical variables: Chi-square or Fisher exact tests

ATS: American Thoracic Society; COPD: chronic obstructive pulmonary disease; HIV: human immunodeficiency virus

Data were missing for ≤ 5% of participants for all covariates other than the following: COPD: 41% (n=500); Asthma: 40% (n=493); HIV: 48% (n=579); Diabetes: 40% (n=492). For these covariates, the above percentage distributions are restricted to participants without missing data.

Table S2. Baseline cohort characteristics among participants in the final analytic cohort* compared to participants not in the final analytic cohort

	Total Cohort (n = 1219)	In analytic cohort (n = 902)	Not in analytic cohort (n = 317)	P value#
**Age**, years	39 [28, 53]	40 [29, 53]	36 [25, 51]	0.006
**Women**	733 (60)	557 (62)	176 (56)	0.051
**Smoking history**				0.421
Never	850 (70)	640 (71)	210 (68)	
Former	242 (20)	175 (20)	67 (22)	
Current	116 (10)	82 (9)	34 (11)	
Years smoked	18 [8, 32]	21 [10, 32]	7 [3, 23]	0.099
**Education**				0.015
None	185 (15)	124 (14)	61 (21)	
Some primary school	390 (33)	299 (33)	91 (31)	
Completed primary school	296 (25)	236 (26)	60 (20)	
At least some secondary school	327 (27)	243 (27)	84 (28)	
**Subsistence farming**	794 (69)	628 (71)	166 (61)	0.002
**Socioeconomic status**				0.085
Poorest	261 (22)	186 (21)	75 (26)	
Poorer	244 (21)	183 (20)	61 (21)	
Middle	233 (20)	182 (20)	51 (18)	
Richer	236 (20)	192 (21)	44 (15)	
Richest	213 (18)	157 (17)	56 (20)	
**Body Mass Index**, kg/m^2				0.0002
Underweight (<18.5)	80 (7)	53 (6)	27 (9)	
Normal (18.5–25)	701 (58)	498 (55)	203 (64)	
Overweight (25.1–30)	305 (25)	235 (26)	70 (22)	
Obese (≥30)	133 (11)	116 (13)	17 (5)	
**Medical Comorbidities**				
COPD	7 (1)	4 (1)	3 (2)	0.386
Asthma	9 (1)	9 (2)	0 (0)	0.121
HIV	34 (5)	25 (5)	9 (6)	0.856
Diabetes	23 (3)	14 (3)	9 (5)	0.145

n (%) or median [interquartile range] unless otherwise specified.

*Defined as participants with ATS-acceptable spirometry and non-missing data on model covariates (age, sex, height, smoking history, occupation, education level, and socioeconomic status).

Continuous variables: Wilcoxon rank sum tests; Categorical variables: Chi-square or Fisher exact tests

ATS: American Thoracic Society; COPD: chronic obstructive pulmonary disease; HIV: human immunodeficiency virus

Data were missing for ≤ 5% of participants for all covariates other than the following: COPD: 41% (n=500); Asthma: 40% (n=493); HIV: 48% (n=579); Diabetes: 40% (n=492). For these covariates, the above percentage distributions are restricted to participants without missing data.

Table S3. Population-weighted correlates of lung function among adults living in rural Uganda (n=902)

	Δ FEV_1 (mL)		Δ FEV_1 (%pred)		Δ FEV_1/FVC		Δ FEV1/FVC (%pred)	
Characteristics	***b***	***95% CI***	***b***	***95% CI***	***b***	***95% CI***	***b***	***95% CI***
Change per year	-10**	-17, -2	0.28	-0.01, 0.56	-0.006***	-0.008, -0.005	-0.53***	-0.68, -0.37
Age, per year	-20***	-22, -18	-0.01	-0.09, 0.06	-0.002***	-0.002, -0.002	-0.02	-0.05, 0.02
Female sex	-559***	-629, -488	-7.45***	-10.23, -4.67	-0.007	-0.018, 0.003	-1.48*	-2.79, -0.16
Ever smoking	-44	-93, 5	-1.82	-3.73, 0.10	-0.010*	-0.018, -0.002	-1.28*	-2.26, -0.30
Height#	32***	28, 37	-0.09	-0.26, 0.08	-0.0001	-0.001, 0.001	0.08	-0.0003, 0.16
Lower socioeconomic status	-22	-61, 18	-0.72	-2.25, 0.81	-0.003	-0.009, 0.004	-0.41	-1.20, 0.37

FEV_1: forced expiratory volume in one second, in milliliters; FVC: forced vital capacity, in milliliters; CI: confidence interval. Reference category for smoking = never smokers and for socioeconomic status = richest quintile of wealth. *$p < 0.05$, **$p < 0.01$, ***$p < 0.001$

Table S4. Population-weighted correlates of lung function among adults living in rural Uganda with normal lung function (n=772)

	Δ FEV_1 (mL)		Δ FEV_1 (%pred)		Δ FEV_1/FVC		Δ FEV1/FVC (%pred)	
Characteristics	***b***	***95% CI***	***b***	***95% CI***	***b***	***95% CI***	***b***	***95% CI***
Change per year	-18***	-25, -11	-0.03	-0.30, 0.24	-0.007***	-0.009, -0.006	-0.66***	-0.80, -0.51
Age, per year	-21***	-22, -19	0.01	-0.05, 0.07	-0.002***	-0.002, -0.002	-0.01	-0.04, 0.02
Female sex	-553***	-616, -490	-6.96***	-9.35, -4.58	-0.010*	-0.019, -0.002	-1.84***	-2.90, -0.78
Ever smoking	-42	-88, 3	-1.89*	-3.64, -0.13	-0.008*	-0.015, -0.001	-1.09*	-1.94, -0.23
Height#	31***	27, 35	-0.22**	-0.37, -0.07	-0.001**	-0.001, -0.0003	-0.003	-0.07, 0.06
Lower socioeconomic status	-18	-54, 18	-0.43	-1.81, 0.96	-0.005	-0.010, 0.001	-0.67	-1.34, 0.01

FEV_1: forced expiratory volume in one second, in milliliters; FVC: forced vital capacity, in milliliters; 95% CI: 95 percent confidence interval; SES: socioeconomic status (dichotomized at the median asset index). *$p < 0.05$, **$p < 0.01$, ***$p < 0.001$

Table S5. Population-weighted lung function decline per year among adults living in rural Uganda from 2015 through 2019, sensitivity analysis utilizing different age cut-offs

	ΔFEV_1 (mL)		ΔFEV_1 (%pred)		ΔFEV_1/FVC		ΔFEV1/FVC (%pred)	
	b	***95% CI***	***b***	***95% CI***	***b***	***95% CI***	***b***	***95% CI***
***Unrestricted adult population (age 18 to 99)**, n=902*								
Change per year	-10**	-17, -2	0.28	-0.01, 0.56	-0.006***	-0.008, -0.005	-0.53***	-0.68, -0.37
***Restricted to participants ≥ 30 years old**, n=711*								
Change per year	-17***	-25, -9	0.13	-0.18, 0.43	-0.007***	-0.008, -0.005	-0.62***	-0.80, -0.44
***Restricted to participants ≥ 40 years old**, n=500*								
Change per year	-20***	-30, -11	0.04	-0.35, 0.42	-0.007***	-0.009, -0.005	-0.68***	-0.92, -0.44

FEV_1: forced expiratory volume in one second, in milliliters; FVC: forced vital capacity, in milliliters; CI: confidence interval. Reference category for smoking = never smokers and for socioeconomic status = richest quintile of wealth. *$p < 0.05$, **$p < 0.01$, ***$p < 0.001$

Figure S1. Health screening locations in southwestern Uganda

The red dots are individual health screening event locations. The map of Uganda is courtesy of Google Maps and the map of Nya kabare Parish, and health screening event locations is courtesy of Mr. Justin Rasmussen, 2017. This image is reproduced with permission from the Journal of Global Health, J Glob Health 2019 Jun;9(1):010434. doi: 10.7189/jogh.09.010434

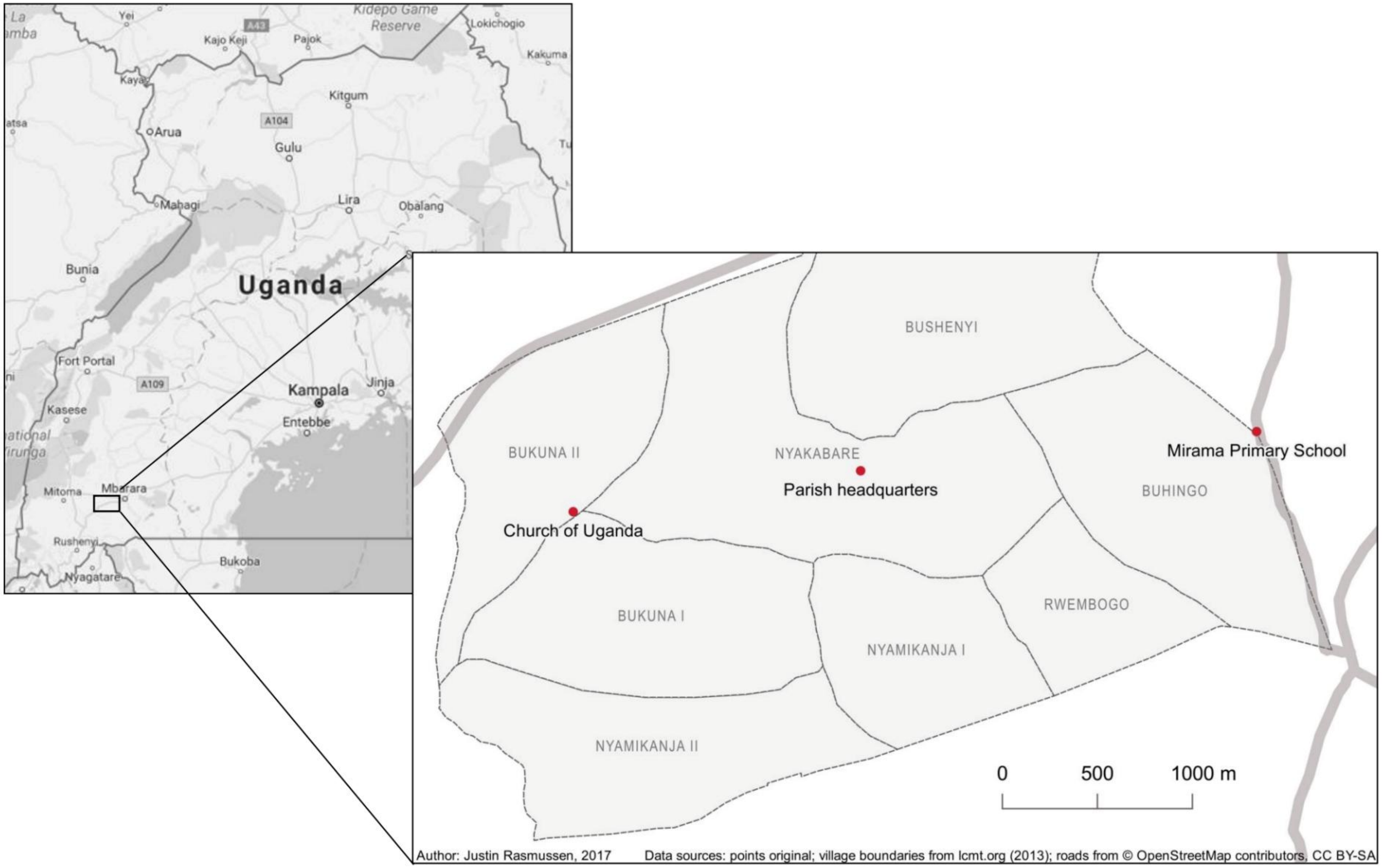

Figure S2. Flow diagram of study participant selection.

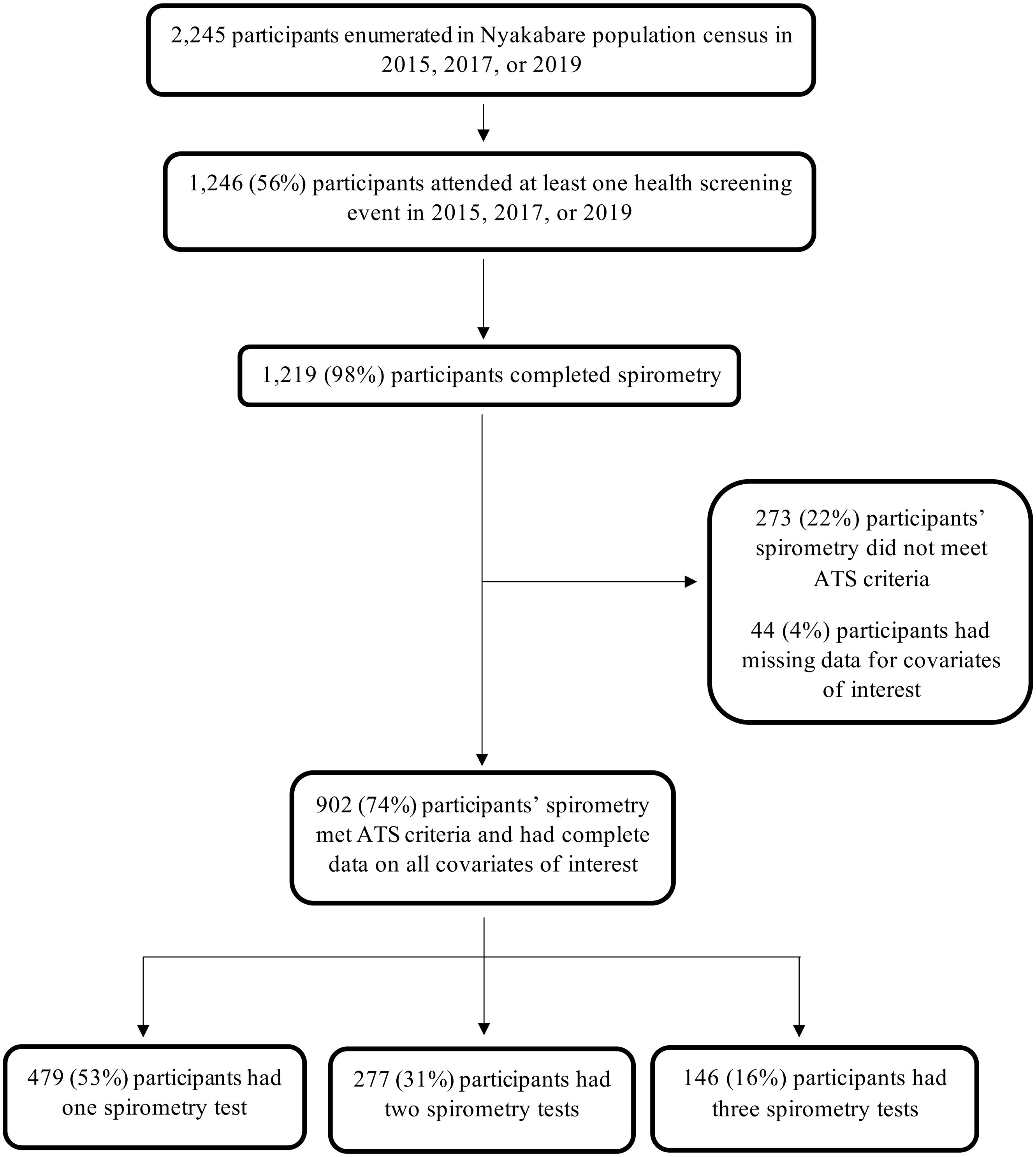

Figure 3, Panel A. Flow diagram of inverse probability of treatment weighting (IPTW) numerator selection – Nyakabare parish adults who attended health screening events in 2015, 2017, or 2019.

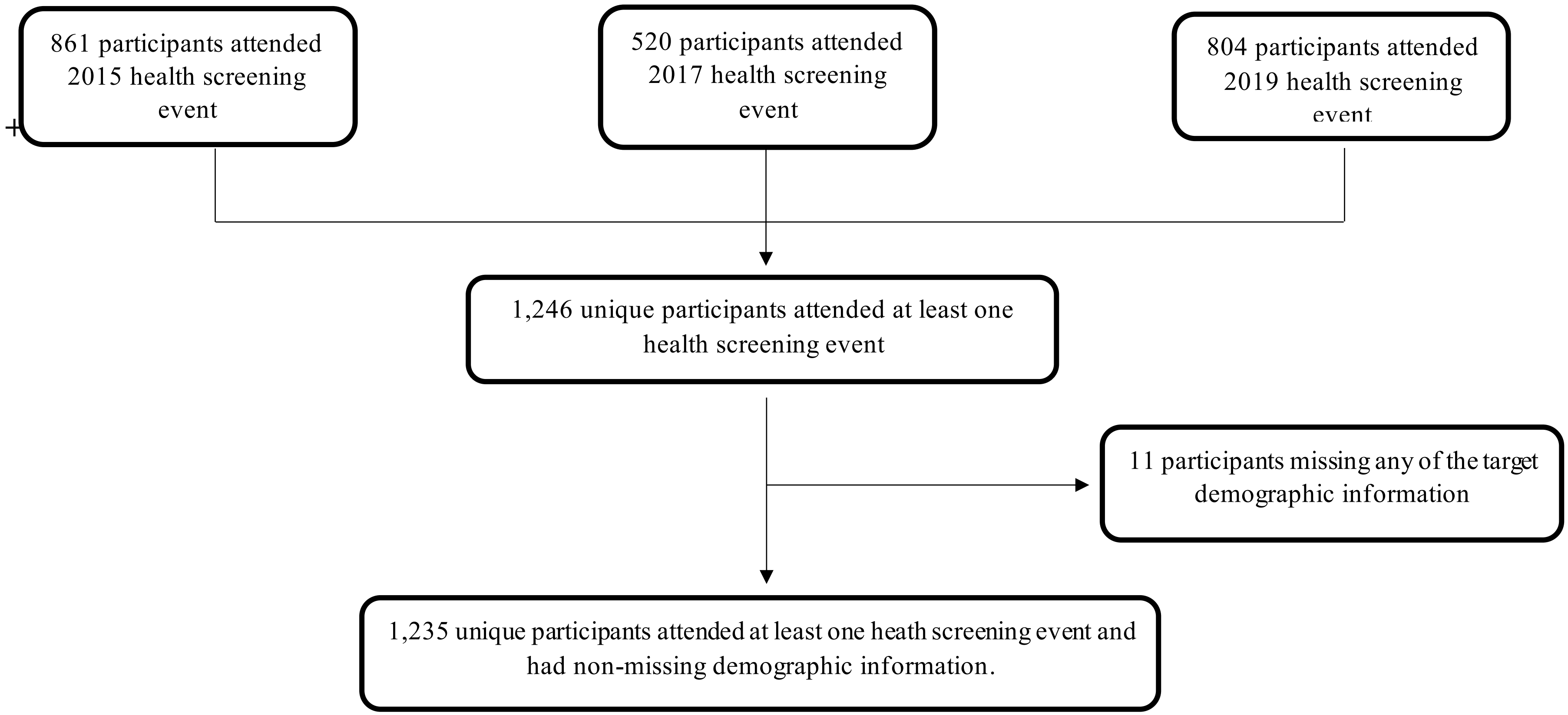

Figure 3, Panel B. Flow diagram of inverse probability of treatment weighting (IPTW) denominator selection – Nyakabare parish adults who were included in the population census in 2015, 2017, or 2019.

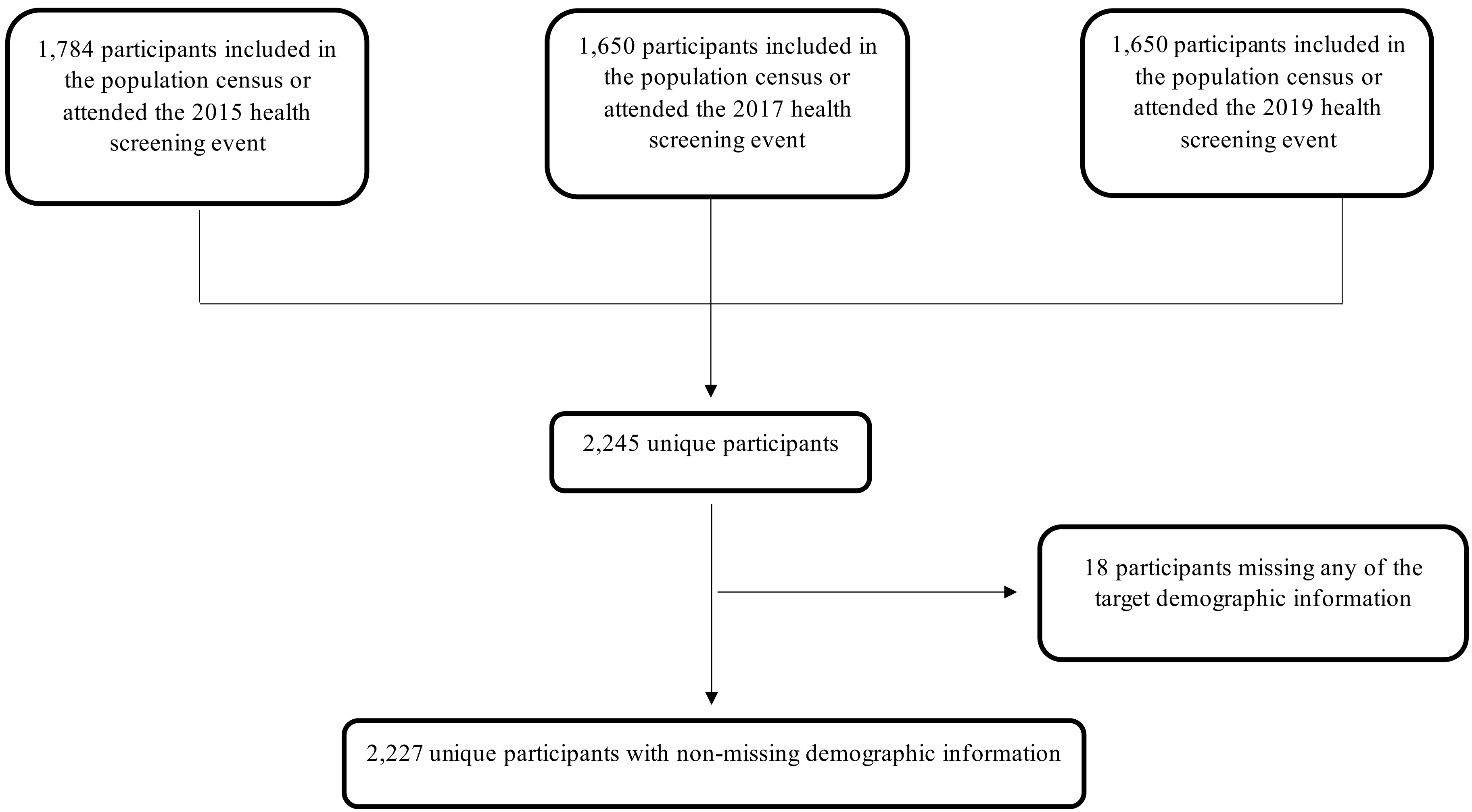

Figure S4. Model diagnostics and fit for primary IPT-weighted linear mixed-effects models

For the primary IPT-weighted linear mixed-effects models, we assessed model convergence and residual diagnostics. All models converged successfully. Residual-versus-fitted plots and quantile–quantile plots of model residuals were reviewed and did not show substantial departures from homoscedasticity or approximate normality. The mixed-effects framework accommodated the unbalanced longitudinal structure, allowing participants to contribute between one and three ATS/ERS-acceptable spirometry observations. Model fit statistics and variance component estimates were reviewed and were consistent with the use of random intercepts to account for within-participant correlation. Diagnostic plots for stratified and sensitivity models showed similar patterns as shown below.

Outcome	Residuals vs fitted	Q-Q plot
**ΔFEV_1 (mL)**	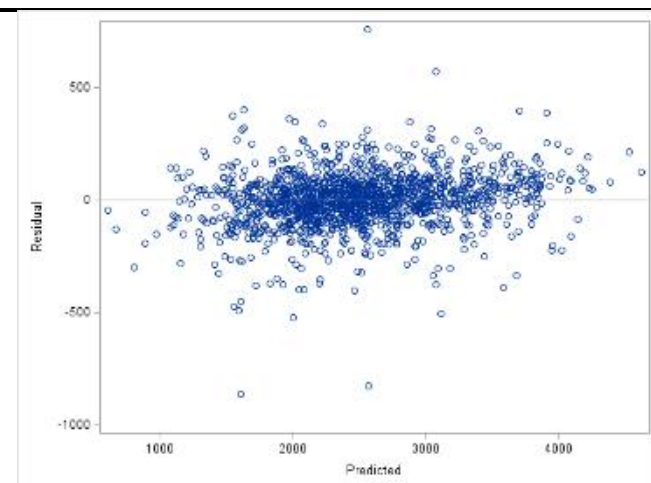	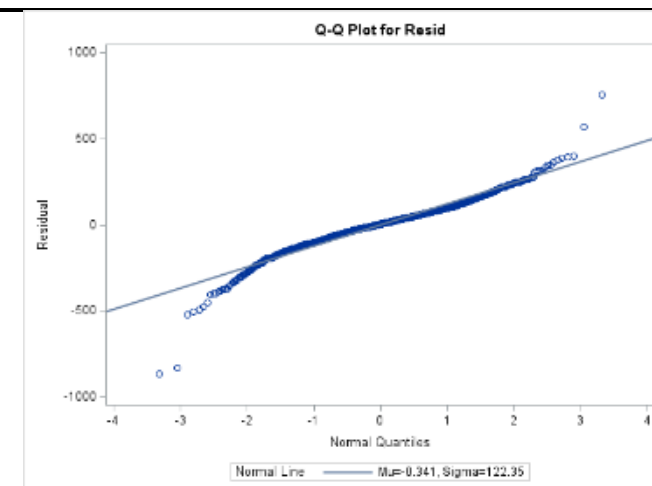
**ΔFEV_1 (%pred)**	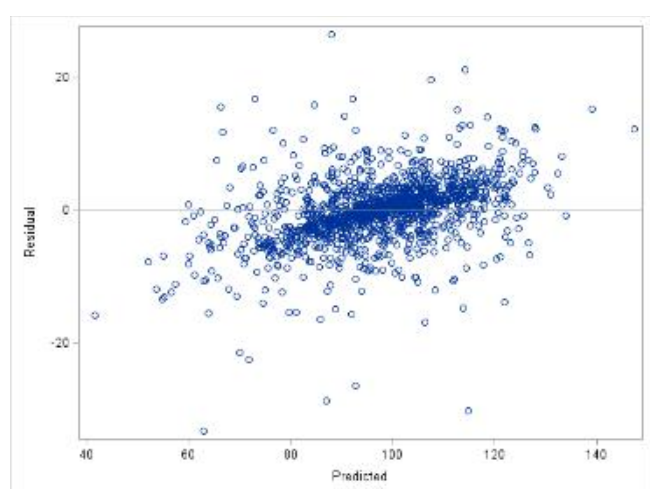	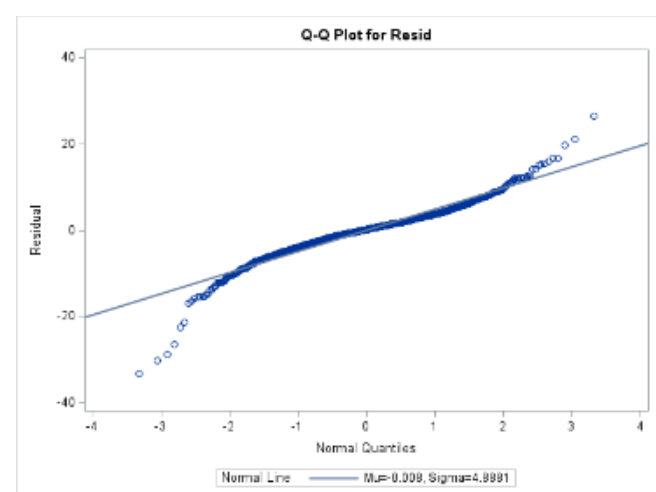
**$\Delta FEV_1/FVC$**	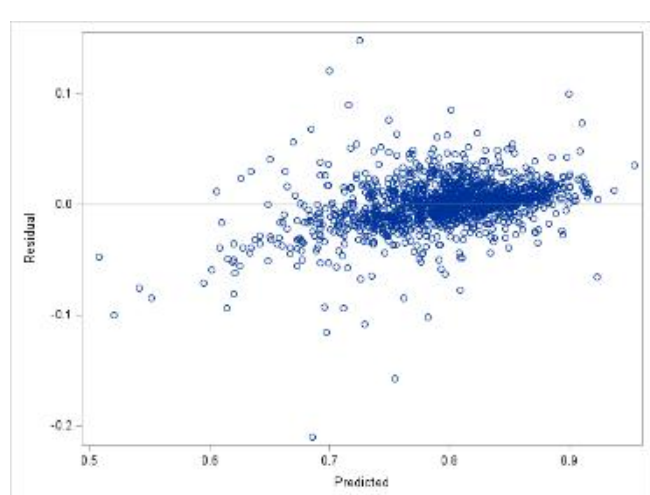	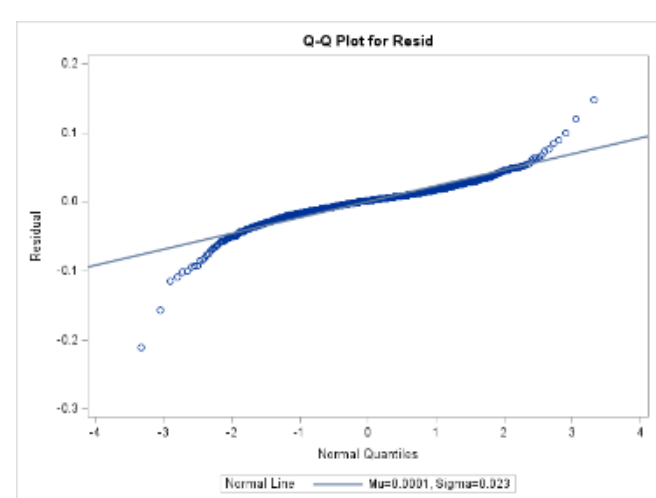
**ΔFEV1/FVC (%pred)**	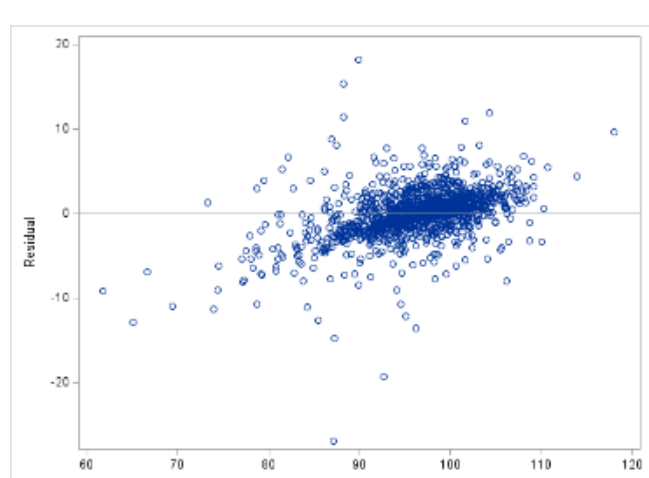	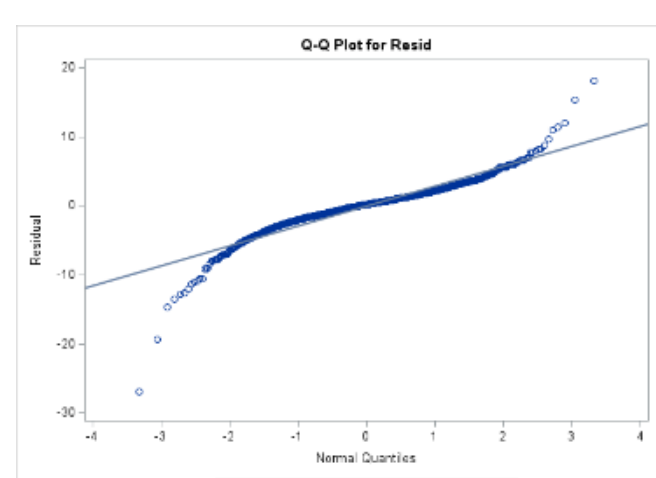
